# Modeling wave scattering in impedance-lined duct networks with expansion chambers

**DOI:** 10.1371/journal.pone.0347139

**Published:** 2026-04-15

**Authors:** Abdulwahed Alrashdi, Naif Alkuhayli, Muhammad Safdar

**Affiliations:** 1 Mathematics Department, College of Science, Jouf University, Sakaka, Saudi Arabia; 2 Department of Mathematics, Quaid-i-Azam University, Islamabad, Pakistan; COMSATS University Islamabad, PAKISTAN

## Abstract

This article presents a general framework for predicting acoustic wave scattering in ducted systems with expansion chambers and dissipative linings, as commonly encountered in engineering noise-control devices. The method represents the acoustic field in each duct segment through orthogonal modal expansions consistent with the local boundary conditions and enforces continuity conditions at the interfaces to determine the reflected and transmitted wave content. Model verification is carried out using representative benchmark configurations excited by a piston-type source. The results show that acoustic linings substantially impact propagation and resonance behavior through changes in modal wavenumbers and coupling between modes. Parametric comparisons between single- and double-lined expansion chamber designs demonstrate that introducing an additional lined cavity strengthens modal interactions, shifts resonance features, and improves attenuation over a broader frequency range. The proposed formulation is computationally efficient, physically interpretable, and readily adaptable to practical duct geometries and lining specifications, making it suitable for the design and optimization of waveguide-based noise mitigation components in applications such as HVAC networks, exhaust systems, and aero-engine ducting.

## 1 Introduction

The study of sound propagation in ducts is crucial in various engineering applications, including noise control in aerospace, automotive, and industrial systems [1–4]. Understanding how sound waves behave in confined spaces, especially those with varying geometries or impedance properties, is essential for designing effective acoustic treatments and predicting noise levels. The presence of impedance liners and cavities induces complex interactions, leading to modal conversion, resonant scattering, and significant transmission loss. Over the past few decades, extensive research has been devoted to modeling sound propagation in ducts due to its importance in various engineering applications. Initial studies largely focused on uniform ducts with simple boundary conditions, enabling analytical treatment. However, real-world systems often involve complex geometries such as bends [5], non-uniform cross sections [6,7], and junctions with extended ducts [8], all of which introduce modal scattering and coupling effects. In addition, the presence of sound-absorbing materials, commonly referred to as liners, plays a crucial role in attenuating acoustic energy. These liners may be locally or non-locally reacting and can exhibit azimuthal segmentation, particularly in cylindrical configurations, which further complicates the wave dynamics [9]. Modeling such materials requires sophisticated approaches capable of capturing spatial impedance variations and multimodal interactions, as discussed in works like [10]. As a result, modern duct acoustics has advanced to include a variety of analytical and numerical methods that account for these geometric and material complexities to improve the prediction and control of sound behavior in practical systems.

Various analytical and numerical methods have been developed to address the complex phenomena associated with sound propagation in ducts featuring non-uniform geometries and boundary conditions. For instance, the multimodal method has emerged as a robust and versatile tool for analyzing wave behavior in non-uniform lined ducts [6,7] and ducts with rigid bends [5], where it effectively captures mode coupling and scattering induced by geometrical variations. For cases involving circumferentially segmented liners, the point matching method has been successfully applied to account for azimuthal discontinuities in impedance and their effects on mode interactions [9,11–16]. Nevertheless, the axial and radial non-uniformities in acoustic liners contain a significant impact on modal characteristics and wave attenuation behavior [17–21]. The wave propagation in systems with slowly varying parameters, such as in ducts with gradually changing cross sections or material properties has been discussed in [22,23]. This framework has further been extended to the study of laser-generated guided elastic waves in hollow cylinders, highlighting its applicability beyond traditional acoustic domains [24].

In parallel with these forward-propagation models, multimodal formulations have also been embedded in impedance-eduction and inverse-design strategies for acoustic liners. Three-dimensional straightforward and mirror-based multimodal methods have been developed to educe liner impedance in grazing-flow ducts and zigzag-array configurations [25–27]. More recently, a fully three-dimensional multimodal inverse method has been proposed that retrieves liner impedance from the measured scattering matrix in a large duct with many cut-on modes [28]. These studies underline the importance of exploiting multi-mode information when characterising and optimising advanced liner configurations, and they further motivate the multimodal scattering framework adopted in the present work. On the other hand, at high frequencies, especially in turbofan engine ducts, the modal structure includes both traditional acoustic modes and surface waves that are strongly influenced by wall impedance and mean flow [29]. In certain configurations, particularly in impedance-walled waveguides, the dispersion relation may exhibit multiple roots. This leads to eigenfunctions that deviate from classical solutions, incorporating linear spatial terms alongside exponential or trigonometric components [30]. To further enhance acoustic attenuation, periodic arrangements of side-branch Helmholtz resonators have been investigated. These systems exploit wave coupling to generate stop bands, thereby broadening the effective attenuation bandwidth beyond that of a single resonator [31]. The present article focused on modeling double liner cavities in acoustic waveguide by using the multimodal method. Recently, the viscous damping effects on wave propagation [32] and acoustic scattering in flexible waveguides with discontinuities [33–38] have been presented. These studies were focused on the consideration of single liner cavity with different medium, material and geometric variation. Despite the extensive research, the accurate and efficient prediction of sound propagation in complex duct systems involving double liner cavities, particularly those with combined geometric variations and advanced acoustic linings, continues to be an area of active research.

The present work is concerned with the theoretical investigation of acoustic wave propagation in a duct lined with locally reacting surfaces. The model assumes a locally reacting liner, no mean flow, and a two-dimensional rectangular waveguide, defining the range of applicability of the results. A multimodal framework is developed to analyze the scattering behavior of incident propagating modes interacting with different boundary configurations, including rigid walls and impedance surfaces. The wave dynamics are governed by the eigenstructure of a transformed system derived from the Helmholtz equation, subject to appropriate boundary conditions. To validate the approach, two benchmark cases are considered: one involving a rigid-walled duct excited by a piston source and another featuring an impedance-lined wall. In both scenarios, the multimodal results are compared with those obtained via the mode-matching technique to confirm accuracy. The mode-matching technique which relies on the properties of the eigenfunctions has been employed for various wave scattering problems, for instance see [39–41]. Building upon these benchmark cases, the study further explores the acoustic behavior of more complex systems involving compliant linings. Specifically, two cavity configurations-single and double lined chambers are examined to assess the impact of wall impedance and structural arrangement on wave attenuation and transmission. The formulation captures the effects of geometric features, excitation frequency, and boundary stiffness on mode coupling and energy dissipation. Through a combination of modal analysis and orthogonality-based techniques, the study offers insights relevant to the design and optimization of noise control systems employing reactive or dissipative linings. The analytical framework developed in this work builds on existing multimodal and mode-matching formulations for lined and non-uniform ducts, such as those reported in [5–7]. The present contribution extends these models in three main directions. First, we explicitly incorporate scattering from a baffled piston source and compare the resulting fields with a standard mode-matching formulation in uniform and singly lined ducts, thereby providing a direct theoretical validation of the approach. Second, we generalize the configuration, which focus on a rigid lower wall and a lined upper wall, by allowing liner–cavity conditions to be imposed on both the lower and upper boundaries. This enables the analysis of more general symmetric and asymmetric lining arrangements within the same analytical setting. Third, and most importantly, we derive and implement a multimodal mode-matching solution for a waveguide containing *two* lined expansion chambers. This double-cavity configuration reveals how an additional lined section modifies the eigenvalue trajectories, alters the resonance structure, and broadens the effective attenuation bandwidth compared with the canonical single-cavity case.

The structure of the article is organized as follows: Section 1 provides the introduction and outlines the motivation for the study. Section 2 presents the mathematical formulation of wave propagation in rigid and impedance-lined ducts excited by a plane piston. Section 3 details the mathematical modeling of acoustic scattering in waveguides containing single and double liner cavities. Section 4 contains the numerical results and analysis. Finally, Section 5 summarizes the key findings and provides concluding remarks.

## 2 Wave propagation in rigid and lined duct radiated by plane

**Fig 1:**
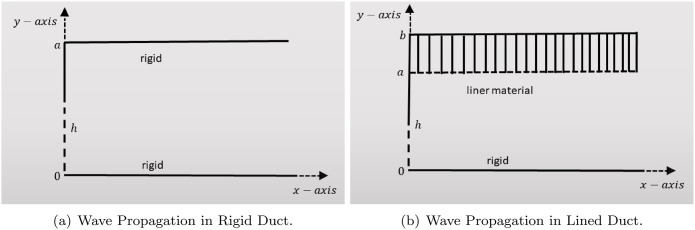
Wave Propagation in Rigid and Lined Duct Radiated by Plane Piston.

This section discusses the propagation of acoustic waves generated by a plane piston located within a duct system composed of both rigid and lined segments. We consider wave motion in a two-dimensional rectangular duct bounded between $\bar{y}=\bar{0}$ and $\bar{y}=\{\bar{a},\bar{b}\}$ in the $\bar{x}\bar{y}$-plane, as illustrated in Fig 1. Fig 1 represents a rigid waveguide with an L-shaped geometry comprising two perpendicular rigid segments. The lower horizontal segment lies along the $\bar{x}$-axis, extending from the origin $\left(\bar{0},\bar{0}\right)$ toward $\bar{x}\to \infty$, labeled as $\bar{0}\leq \bar{x}\leq \infty$. A vertical segment rises from the origin to a height $\bar{h}$, marked with a dashed line, covering the interval $\bar{0}\leq \bar{y}\leq \bar{h}$. From the point $\bar{y}=\bar{a}$, another rigid horizontal segment extends in the positive $\bar{x}$-direction, denoted as $\bar{a}\leq \bar{x}\leq \infty$, and runs parallel to the lower horizontal segment. All boundaries shown in this configuration are rigid. In contrast, Fig 1b illustrates a lined acoustic duct configuration. Here, the lower boundary between $\bar{y}=\bar{0}$ and $\bar{y}=\bar{h}$ is acoustically non-rigid (represented by dashed lines), while the segment from $\bar{y}=\bar{h}$ to $\bar{y}=\bar{b}$ is rigid. The section from $\bar{y}=\bar{a}$ to $\bar{y}=\bar{b}$ is lined with an acoustic material. The upper horizontal boundary $\bar{b}\leq \bar{x}\leq \infty$ and the lower horizontal boundary $\bar{0}\leq \bar{x}\leq \infty$ define the duct’s length in the $\bar{x}$-direction and are parallel. The entire duct is filled with a compressible fluid medium characterized by a constant density ρ and a sound speed *c*, through which acoustic waves propagate. The acoustic pressure $\bar{P}(\bar{x},\bar{y},\bar{t})$ satisfies the dimensional wave equation:


$$\nabla^{2}\bar{P}(\bar{x},\bar{y},\bar{t})=\frac{1}{{\bar{c}}^{2}}\frac{\partial^{2}\bar{P}}{\partial {\bar{t}}^{2}}.$$ (1)

Assuming a harmonic time dependence of the form $e^{-i\bar{\omega }\bar{t}}$, where $\bar{\omega }$ denotes the angular frequency, the pressure field can be written as:


$$\bar{P}(\bar{x},\bar{y},\bar{t})=\bar{p}(\bar{x},\bar{y})e^{-i\bar{\omega }\bar{t}}.$$ (2)

The duct boundaries can be either rigid or lined with an impedance surface. For rigid boundaries, the normal component of the velocity must vanish:


$$\hat{n}\cdot \bar{V}=0,$$ (3)

where $\hat{n}$ is the unit normal vector directed into the surface. The pressure $\bar{P}$ and velocity $\bar{V}$ are related via the momentum equation:


$$\frac{\partial \bar{V}}{\partial \bar{t}}=-\nabla \bar{P}.$$

Using the assumed harmonic time dependence, the velocity field is expressed as:


$$\bar{V}(\bar{x},\bar{y},\bar{t})=\bar{v}(\bar{x},\bar{y})e^{-i\bar{\omega }\bar{t}}.$$ (4)

To nondimensionalize the equations, we introduce the following transformations:


$$x=\frac{\bar{x}}{\bar{H}},\;y=\frac{\bar{y}}{\bar{H}},\;p=\frac{\bar{p}}{\rho {\bar{c}}^{2}},$$ (5)

where $\bar{H}$ is a characteristic length and ρ is the fluid density. Substituting (2) into (1) and using the transformations in (5), the nondimensional form of the Helmholtz equation becomes:


$$\left(\frac{\partial^{2}}{\partial x^{2}}+\frac{\partial^{2}}{\partial y^{2}}+K^{2}\right)p(x,y)=0,$$ (6)

subject to the rigid boundary condition:


$$\hat{n}\cdot \nabla p=0,$$ (7)

where $K=\frac{\bar{\omega }\bar{H}}{\bar{c}}$ is the nondimensional wave number or frequency parameter.

### 2.1 Wave propagation in rigid duct radiated by plane piston

The acoustic wave propagation in a rigid-walled duct driven by a plane piston source is illustrated in Fig 1. In the following section, we employ the multimodal method to establish the mathematical formulation of the problem.

#### 2.1.1 Multimodal solution.

Consider the propagation of incident acoustic waves in a duct along the positive *x* -direction. The acoustic pressure field within the guiding channel can be expressed in terms of a modal expansion as:


$$p(x,y)=\sum_{n=0}^{\infty }A_{n}\psi_{n}(y)e^{i\eta_{n}x},$$ (8)

where $\psi_{n}(y)$ denotes the transverse eigenfunction corresponding to the *n*^th^ mode, $\eta_{n}$ is the axial wavenumber, and *A*_*n*_ represents the mode amplitude. These quantities are initially unknown and will be determined using a multimodal approach. From Equation (7), the dimensionless rigid-wall boundary conditions associated with the problem are:


$$\frac{\partial p}{\partial y}=0,\;y\in \{0,a\},\;x>0,$$ (9)

$$\frac{\partial p}{\partial x}=0,\;x=0,\;h\leq y\leq a.$$ (10)

Substituting the modal representation (8) into the governing equation (6) and applying the boundary conditions (9)–(10), we arrive at the following ordinary differential equation for the mode shapes:


$$\left(\frac{d^{2}}{dy^{2}}+\gamma_{n}^{2}\right)\psi_{n}(y)=0,$$ (11)

subject to the Neumann-type boundary conditions:


$$\psi_{n}^{\prime }(y)=0,\;y\in \{0,a\},$$ (12)

where $\gamma_{n}=\sqrt{K^{2}-\eta_{n}^{2}}$, and the prime denotes differentiation with respect to *y*. To determine the admissible solutions to the system defined by (11)–(12), we proceed by formulating the corresponding eigenvalue problem. The transverse eigenvalue problem is defined as:


$$\frac{d^{2}\xi_{n}(y)}{dy^{2}}+\alpha_{n}^{2}\xi_{n}(y)=0,$$ (13)

subject to the Neumann boundary conditions:


$$\xi_{n}^{\prime }(y)=0,\;y\in \{0,a\}.$$ (14)

Solving Equation (13) under the boundary conditions (14) yields the orthonormal eigenfunctions:


$$\xi_{n}(y)=\Lambda_{n}\cos(\alpha_{n}y),\;n=0,1,2,\ldots ,$$

with corresponding eigenvalues:


$$\alpha_{n}=\frac{n\pi }{a},\;n=0,1,2,\ldots .$$

These eigenfunctions satisfy the orthonormality condition:


$$\int_{0}^{a}\xi_{n}(y)\;\xi_{m}(y)\;dy=\delta_{mn},$$ (15)

where $\delta_{mn}$ is the Kronecker delta, defined as:


$$\begin{array}{c} \delta_{mn}=\left\{ \begin{array}{cc} 1, & \text{if }m=n,\\ 0, & \text{if }m\neq n. \end{array}\right. \end{array}$$

The functions $\{\xi_{m}(y){\}}_{m\geq 0}$ thus form a complete orthonormal basis of rigid-duct modes. In all subsequent developments, including the lined sections, the transverse dependence of the acoustic field is represented in this common basis. To construct a solution to the system defined by Equations (11)–(12), we expand $\psi_{n}(y)$ in terms of the orthonormal eigenfunctions:


$$\psi_{n}(y)=\sum_{m=0}^{\infty }B_{nm}\;\xi_{nm}(y)=\xi^{t}B,$$ (16)

where


$$\begin{array}{c} \xi^{t}=(\begin{array}{ccccc} \xi_{n0} & \xi_{n1} & \cdots & \xi_{nm} & \cdots \end{array}),\;B=(\begin{array}{c} B_{n0}\\ B_{n1}\\ \vdots \\ B_{nm}\\ \vdots \end{array}). \end{array}$$

Here, the superscript *t* indicates the transpose of the vector ξ. Multiplying Equation (11) by ξ and integrating over the interval y∈[0,a], we obtain:


$$\int_{0}^{a}\xi \;\frac{d^{2}\psi_{n}}{dy^{2}}\;dy+\gamma_{n}^{2}\int_{0}^{a}\xi \;\psi_{n}\;dy=0.$$ (17)

The first integral in Equation (17) is evaluated using integration by parts. Applying the boundary conditions (12) and (14), we find:


$$\int_{0}^{a}\xi \;\frac{d^{2}\psi_{n}}{dy^{2}}\;dy=\int_{0}^{a}\psi_{n}(y)\;\frac{d^{2}\xi }{dy^{2}}\;dy.$$ (18)

Substituting Equation (18) into Equation (17) gives:


$$\int_{0}^{a}\psi_{n}(y)\;\frac{d^{2}\xi }{dy^{2}}\;dy+\gamma_{n}^{2}\int_{0}^{a}\xi (y)\;\psi_{n}(y)\;dy=0.$$ (19)

Now, substituting the eigenfunction expression $\xi_{n}(y)=\Lambda_{n}\cos(\alpha_{n}y)$ and the expansion (16) into Equation (19), and simplifying the resulting expression, we obtain:


$$-{\left(\frac{m\pi }{a}\right)}^{2}\sum_{p=0}^{\infty }B_{np}\;\delta_{pm}+\gamma_{n}^{2}\sum_{p=0}^{\infty }B_{np}\;\delta_{pm}=0,\;m=0,1,2,\ldots .$$ (20)

In matrix form, Equation (20) can be rewritten as:


$$-N_{1}IB+\gamma_{n}^{2}IB=0,$$ (21)

where


$$\begin{array}{c} N_{1}=(\begin{array}{ccccc} 0 & 0 & 0 & \cdots & 0\\ 0 & \frac{\pi^{2}}{a^{2}} & 0 & \cdots & 0\\ 0 & 0 & \frac{4\pi^{2}}{a^{2}} & \cdots & 0\\ \vdots & \vdots & \vdots & \ddots & \vdots \\ 0 & 0 & 0 & \cdots & \frac{m^{2}\pi^{2}}{a^{2}}\\ \vdots & \vdots & \vdots & \vdots & \vdots \end{array}). \end{array}$$

Since $\gamma_{n}^{2}=K^{2}-\eta_{n}^{2}$, Equation (21) becomes:


$$\eta_{n}^{2}B=NB,$$ (22)

where the matrix *N* is defined as:


$$N=K^{2}I-N_{1}.$$

Here, the matrix *N* contains the eigenvalues $\eta_{n}^{2}$ and the eigenvectors form the modal matrix *X*. Consequently, the acoustic pressure field can be expressed as:


$$p(x,y)=\xi^{t}\left[XD(x)A+XD(-x)B\right],$$ (23)

where


$$\begin{array}{c} A^{t}=[\begin{array}{ccccc} A_{0} & A_{1} & \cdots & A_{n} & \cdots \end{array}],\;B^{t}=[\begin{array}{ccccc} \delta_{00} & \delta_{10} & \cdots & \delta_{n0} & \cdots \end{array}], \end{array}$$

and *D*(*x*) is a diagonal matrix given by:


$$\begin{array}{c} D(x)=(\begin{array}{cccc} e^{i\eta_{0}x} & 0 & 0 & \cdots \\ 0 & e^{i\eta_{1}x} & 0 & \cdots \\ 0 & 0 & e^{i\eta_{2}x} & \cdots \\ \vdots & \vdots & \vdots & \ddots \end{array}). \end{array}$$

Here, ***A***^*t*^ and ***B***^*t*^ are the transport coefficient vectors, and *D*(*x*) is the propagation matrix. Now consider that the duct is excited by a rigid piston located along the wall segment 0 ≤ *y* ≤ *h* at *x* = 0, oscillating with constant velocity *U*. This results in the boundary condition:


$${\frac{\partial p}{\partial x}|}_{x=0}=U,\;0\leq y\leq h.$$ (24)

Differentiating Equation (23) with respect to *x* and evaluating at *x* = 0, we match it with the imposed velocity condition:


$$\xi^{t}X{\frac{dD(x)}{dx}|}_{x=0}A+\xi^{t}X{\frac{dD(-x)}{dx}|}_{x=0}B=U,\;0\leq y\leq h.$$ (25)

Multiplying Equation (25) from the left by ξ and integrating over y∈[0,a], we obtain:


$$\int_{0}^{a}\xi \xi^{t}\;dy\cdot X{\frac{dD(x)}{dx}|}_{x=0}A+\int_{0}^{a}\xi \xi^{t}\;dy\cdot X{\frac{dD(-x)}{dx}|}_{x=0}B=U\int_{0}^{h}\xi (y)\;dy.$$ (26)

This can be simplified as:


$$iX{\frac{dD(x)}{dx}|}_{x=0}A-iX{\frac{dD(-x)}{dx}|}_{x=0}B=UQ,$$ (27)

where the vector *Q*_*m*_ has components:


$$\begin{array}{c} Q_{m}=\int_{0}^{h}\xi_{m}(y)\;dy=\left\{ \begin{array}{cc} \frac{h}{\sqrt{a}}, & m=0,\\ \sqrt{\frac{2}{a}}\cdot \frac{a}{m\pi }\sin\left(\frac{m\pi h}{a}\right), & m\geq 1. \end{array}\right. \end{array}$$ (28)

Therefore, the final expression for ***A*** becomes:


$$A=B-iD_{0}^{-1}X^{-1}QU,$$ (29)

where *D*_0_ is the diagonal matrix given by:


$$D_{0}={\frac{dD(x)}{dx}|}_{x=0}.$$

### 2.2 Wave propagation in lined duct radiated by plane piston

Fig 1b illustrates acoustic wave propagation in a duct with a lined upper wall, driven by a plane piston source. In the subsequent section, we apply the liner boundary condition to the compliant wall and employ the multimodal method to derive the mathematical formulation of the problem.

#### 2.2.1 Liner condition.

We consider the propagation of sound in a two-dimensional channel, as illustrated in Fig 1b. The lower wall, located at *y* = 0, is acoustically rigid, whereas the upper wall at *y* = *a* is compliant and characterized by a frequency-dependent admittance Y(ω). Since we assume a purely reactive (non-absorbing) liner in this study, the admittance can be expressed as


$$Y(\omega )=\frac{1}{Z(\omega )},$$

where Z(ω) denotes the acoustic impedance. An analytical expression for the impedance can be derived as follows. In the single layer region between a≤y≤b, the acoustic pressure satisfies the one-dimensional wave equation:


$$\frac{\partial^{2}\bar{P}}{\partial {\bar{y}}^{2}}=\frac{1}{{\bar{c}}^{2}}\frac{\partial^{2}\bar{P}}{\partial {\bar{t}}^{2}}.$$ (30)

Assuming time-harmonic behavior with the factor $e^{-i\omega t}$, the equation simplifies to:


$$\frac{d^{2}p}{dy^{2}}+\omega^{2}p(y)=0,$$ (31)

where $\omega =\frac{2\pi fH}{c}$ is the normalized angular frequency, and all spatial quantities are non-dimensionalized using the waveguide height *H*. The general solution to Equation (31) is:


$$p(y)=c_{1}\cos(\omega y)+c_{2}\sin(\omega y),$$ (32)

where *c*_1_ and *c*_2_ are constants. The normal component of the acoustic velocity, $v=𝐯\cdot \hat{𝐧}$, is given by:


$$v=-\frac{i}{\omega }\frac{dp}{dy}.$$ (33)

Substituting Equation (32) into Equation (33), we obtain:


$$v(y)=\frac{1}{i\omega }\left(-c_{1}\omega \sin(\omega y)+c_{2}\omega \cos(\omega y)\right).$$ (34)

At the rigid backing located at *y* = *b*, the normal velocity must vanish, i.e., *v*(*b*) = 0. Using this condition, and substituting Equations (32) and (34) into the definition of impedance,


$$Z=\frac{p(y)}{v(y)},$$ (35)

we derive the impedance at point *y* as:


Z(ω)=icot[ω(b−y)]. (36)

Therefore, the normalized acoustic admittance of the compliant upper wall at *y* = *a* becomes:


Y(ω)=−itan[ω(b−a)]. (37)

Note that Y(ω) in (37) is purely imaginary and thus represents a lossless, purely reactive wall admittance with no face-sheet resistance. In this setting the liner does not absorb acoustic energy; all attenuation observed later in terms of transmission loss arises from interference and resonance effects rather than from material dissipation. Hence, for a liner wall backed by a rigid cavity at *y* = *b*, the boundary condition at the compliant surface *y* = *a* can be expressed as:


$$\frac{dp}{dy}=i\omega Y(\omega )p(a),\;y=a.$$ (38)

At the rigid lower wall, the boundary condition becomes:


$$\frac{dp}{dy}=0,\;y=0.$$ (39)

#### 2.2.2 Multimodal solution.

By substituting the modal expansion (8) into the governing equation (6), and applying the boundary conditions (38)–(39), we obtain the following differential equation for the transverse mode shape:


$$\left(\frac{d^{2}}{dy^{2}}+\gamma_{n}^{2}\right)\psi_{n}(y)=0,$$ (40)

along with the boundary conditions:


$$\psi_{n}^{\prime }(0)=0,$$ (41)

$$\psi_{n}^{\prime }(a)=i\omega Y(\omega )\psi_{n}(a).$$ (42)

Multiplying Equation (40) by the eigenfunction vector ξ and integrating over the interval y∈[0,a], we obtain:


$$\int_{0}^{a}\xi \frac{d^{2}\psi_{n}}{dy^{2}}\;dy+\gamma_{n}^{2}\int_{0}^{a}\xi \psi_{n}\;dy=0.$$ (43)

To evaluate the first integral in (43), we apply integration by parts. Using the boundary conditions (41)–(42) along with the Neumann conditions for ξ from (14), we arrive at:


$$\int_{0}^{a}\xi \frac{d^{2}\psi_{n}}{dy^{2}}\;dy=i\omega Y(\omega )\psi_{n}(a)\xi +\int_{0}^{a}\psi_{n}(y)\frac{d^{2}\xi }{dy^{2}}\;dy.$$ (44)

Substituting this into (43) yields:


$$i\omega Y(\omega )\psi_{n}(a)\xi +\int_{0}^{a}\psi_{n}(y)\frac{d^{2}\xi }{dy^{2}}\;dy+\gamma_{n}^{2}\int_{0}^{a}\xi \psi_{n}(y)\;dy=0.$$ (45)

Now, substituting the eigenfunction representation $\xi_{n}(y)=\Lambda_{n}\cos(\alpha_{n}y)$ and the modal expansion (16) into (45), and then applying the orthonormality condition (15), we obtain:


$$i\omega Y(\omega )\sum_{p=0}^{\infty }B_{np}\xi_{p}(0)\xi_{m}(0)-{\left(\frac{m\pi }{a}\right)}^{2}\sum_{p=0}^{\infty }B_{np}\delta_{pm}+\gamma_{n}^{2}\sum_{p=0}^{\infty }B_{np}\delta_{pm}=0.$$ (46)

The above relation can be expressed in matrix form as:


$$\gamma_{n}^{2}IB=N_{1}IB-i\omega Y(\omega )MB,$$ (47)

where *N*_1_ is a diagonal matrix with entries $(\pi m/a{)}^{2}$, and *M* is a matrix with components $M_{mn}=\xi_{m}(0)\xi_{n}(0)$. Using the identity $\gamma_{n}^{2}=K^{2}-\eta_{n}^{2}$ and the admittance expression (37), we can rewrite Equation (47) as:


$$\eta_{n}^{2}IB=\left[K^{2}I+\omega \tan(\omega (b-a))M-N_{1}\right]B.$$ (48)

Note that the corresponding lined-duct modes are represented as products of the rigid basis and this eigenvector matrix $\xi^{t}X$. For lined and rigid ducts the values of matrix *X* are different. For rigid duct *X* is an identity matrix. Once the eigenvalue problem (48) is solved to obtain $\eta_{n}$ and ***B***, the remaining steps to determine the modal amplitudes ***A*** follow the same procedure outlined in Equations (23)–(29), using the boundary condition (24), which describes a rigid piston located along the wall segment 0≤y≤h at *x* = 0, oscillating with constant velocity.

## 3 Acoustic scattering in waveguides with single and double liner cavities

**Fig 2:**
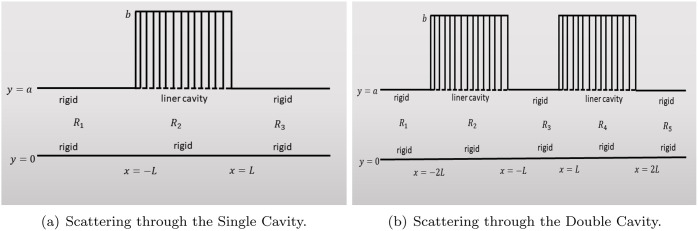
Acoustic Wave Propagation and Scattering in Waveguides with Single and Double Liner Cavities.

In this section, we examine two acoustic waveguide configurations featuring single and double liner cavities, as illustrated in Fig 2. The schematic in Fig 2a depicts a two-dimensional duct system with a partially lined upper wall. The duct extends along the horizontal $\bar{x}$-axis and is divided into three regions: Region *R*_1_, where $\bar{x}<-\bar{L}$; Region *R*_2_, where $|\bar{x}|⩽\bar{L}$; and Region *R*_3_, where $\bar{x}>\bar{L}$. The lower boundary at $\bar{y}=0$ is rigid throughout the entire domain. The upper boundary at $\bar{y}=\bar{a}$ is rigid in Regions *R*_1_ and *R*_3_, while it becomes compliant in the central Region *R*_2_ due to the presence of a liner backed by a rigid cavity, which extends vertically from $\bar{y}=\bar{a}$ to $\bar{y}=\bar{b}$. In this setup, Region *R*_1_ functions as the inlet containing incident and reflected fields, Region *R*_3_ acts as the outlet with only outgoing waves, and Region *R*_2_ serves as the lined section where acoustic attenuation occurs due to the locally reacting liner. Fig 2b presents a configuration with two liner cavities embedded in a waveguide comprising alternating rigid and compliant segments along the upper boundary at $\bar{y}=\bar{a}$, while the lower wall at $\bar{y}=0$ remains entirely rigid. The waveguide is partitioned into five regions along the $\bar{x}$-axis. Region *R*_1_, the inlet region, extends from $\bar{x}<-2\bar{L}$ and is bounded by rigid walls on both the upper and lower sides. Region *R*_2_, defined for $-2\bar{L}<\bar{x}<-\bar{L}$, features a rigid lower wall and a compliant upper wall with a liner segment extending from $\bar{y}=\bar{a}$ to $\bar{y}=\bar{b}$. Region *R*_3_, between $\bar{x}=-\bar{L}$ and $\bar{x}=\bar{L}$, has rigid boundaries on both the upper and lower surfaces and serves as the central rigid segment. Region *R*_4_, spanning $\bar{L}<\bar{x}<2\bar{L}$, mirrors Region *R*_2_ with a liner cavity on the upper boundary. Finally, Region *R*_5_, where $\bar{x}>2\bar{L}$, is the outlet region and is fully rigid on both walls. This double-cavity arrangement represents a periodic acoustic duct with localized compliance, allowing for the investigation of how such liner configurations affect the propagation of sound. These liner segments can introduce complex wave phenomena such as scattering, attenuation, and mode conversion. The entire duct is filled with a compressible fluid characterized by density ρ and sound speed *c*, enabling the analysis of acoustic wave behavior under different boundary conditions and cavity configurations.

### 3.1 Scattering through single lined cavity

We begin by formulating the mathematical model for the single lined cavity configuration, as depicted in Fig 2a. In this setup, the dimensionless acoustic pressure in the duct is represented piecewise across three distinct regions:


$$\begin{array}{c} p(x,y)=\left\{ \begin{array}{cc} p_{1}(x,y), & \;x\leq -L,\;0\leq y\leq a,\\ p_{2}(x,y), & \;-L\leq x\leq L,\;0\leq y\leq b,\\ p_{3}(x,y), & \;x\geq L,\;0\leq y\leq a. \end{array}\right. \end{array}$$ (49)

The corresponding boundary conditions in dimensional form are specified as follows. In Region 1, both upper and lower walls are rigid, hence:


$$\frac{\partial p_{1}}{\partial y}=0,\;y=0,\;a,\;x\leq -L.$$ (50)

In Region 2, the lower wall remains rigid while the upper wall is compliant and modeled by an acoustic liner. Thus, the boundary conditions become:


$$\frac{\partial p_{2}}{\partial y}=0,\;y=0,\;-L\leq x\leq L,$$ (51)

$${\frac{\partial p_{2}}{\partial y}|}_{y=a}=i\omega Y(\omega )p_{2}(y),\;-L\leq x\leq L.$$ (52)

Finally, in Region 3, the duct walls are again rigid, leading to the boundary condition:


$$\frac{\partial p_{3}}{\partial y}=0,\;y=0,\;a,\;x\geq L.$$ (53)

In the following section, the multimodal method is employed to obtain an analytical solution for the acoustic pressure field in this configuration.

#### 3.1.1 Multimodal solution.

As established in Section 2.2.2, we previously derived the mathematical formulation for the duct with a lined upper wall. In this section, we follow a similar approach to analyze the single lined cavity configuration shown in Fig 2a. The relevant equations from the earlier formulation, namely equations (40)–(48), are applied here. We now derive the boundary conditions for the single liner duct and subsequently incorporate both the liner boundary condition and the pressure field formulation for this configuration. The acoustic pressure in each region of the duct is expressed as a linear combination of transverse modes. Specifically, the modal expansions in Regions 1, 2, and 3 are given by:


$$p_{1}(x,y)=\sum_{n=0}^{\infty }A_{1n}\psi_{n}(y)e^{is_{n}(x+L)}+\sum_{n=0}^{\infty }B_{1n}\psi_{n}(y)e^{-is_{n}(x+L)},$$ (54)

$$p_{2}(x,y)=\sum_{n=0}^{\infty }A_{2n}\psi_{n}(y)e^{i\eta_{n}x}+\sum_{n=0}^{\infty }B_{2n}\psi_{n}(y)e^{-i\eta_{n}x},$$ (55)

$$p_{3}(x,y)=\sum_{n=0}^{\infty }A_{3n}\psi_{n}(y)e^{is_{n}(x-L)}+\sum_{n=0}^{\infty }B_{3n}\psi_{n}(y)e^{-is_{n}(x-L)}.$$ (56)

These expressions can be compactly written in matrix form by using (16) along with appropriate amplitude as:


$$p_{1}=\xi^{t}\left[D_{1}(x+L)A_{1}+D_{1}(-x-L)B_{1}\right],$$ (57)

$$p_{2}=\xi^{t}X\left[D_{2}(x)A_{2}+D_{2}(-x)B_{2}\right],$$ (58)

$$p_{3}=\xi^{t}\left[D_{1}(x-L)A_{3}+D_{1}(-x+L)B_{3}\right].$$ (59)

In the matrix formulation (57)–(59) these functions are consistently re-expressed in the common rigid-duct basis $\left\{\xi_{m}\right\}$ using the transformation defined in Section 2.2, with the lined region involving the modal matrix *X*.

Here, the vectors ***A***_1_, ***A***_2_, ***A***_3_ and ***B***_1_, ***B***_2_, ***B***_3_ represent the unknown modal coefficients corresponding to the forward- and backward-propagating modes in each region. The matrices *D*_1_(*x*) and *D*_2_(*x*) are diagonal, with exponential terms describing the axial variation of modes. Specifically, *D*_1_(*x*) is defined using the axial wavenumber $s_{n}=\sqrt{K^{2}-\alpha_{n}^{2}}$ for regions bounded by rigid walls, while *D*_2_(*x*) is defined using the modified axial wavenumber $\eta_{n}$ in the lined region.


$$D_{1}(x)=\text{diag}\left(e^{is_{0}x},e^{is_{1}x},e^{is_{2}x},\ldots \right),\;D_{2}(x)=\text{diag}\left(e^{i\eta_{0}x},e^{i\eta_{1}x},e^{i\eta_{2}x},\ldots \right).$$

These modal expressions and matrix representations form the foundation for applying boundary and continuity conditions in order to solve the acoustic scattering problem in the single liner duct. To determine the unknown modal coefficients, we enforce continuity conditions on both the acoustic pressure and the normal particle velocity at the interfaces *x* = −*L* and *x* = *L*. The continuity of pressure across these interfaces yields:


$$p_{1}(-L,y)=p_{2}(-L,y),\;0\leq y\leq b,$$ (60)

$$p_{2}(L,y)=p_{3}(L,y),\;0\leq y\leq b.$$ (61)

Similarly, continuity of the normal velocity (proportional to the pressure gradient in the *x* -direction) leads to:


$${\frac{\partial p_{1}}{\partial x}|}_{x=-L}={\frac{\partial p_{2}}{\partial x}|}_{x=-L},\;0\leq y\leq b,$$ (62)

$${\frac{\partial p_{2}}{\partial x}|}_{x=L}={\frac{\partial p_{3}}{\partial x}|}_{x=L},\;0\leq y\leq b.$$ (63)

Substituting the modal expansions from equations (57)–(59) into the continuity conditions (60)–(63), we obtain the following matrix equations:


$$\xi^{t}\left[D_{1}(0)A_{1}+D_{1}(0)B_{1}\right]=\xi^{t}X\left[D_{2}(-L)A_{2}+D_{2}(L)B_{2}\right],$$ (64)

$$\xi^{t}X\left[D_{2}(L)A_{2}+D_{2}(-L)B_{2}\right]=\xi^{t}\left[D_{1}(0)A_{3}+D_{1}(0)B_{3}\right],$$ (65)

$$\xi^{t}K_{R}\left[D_{1}(0)A_{1}-D_{1}(0)B_{1}\right]=\xi^{t}XK_{Y}\left[D_{2}(-L)A_{2}-D_{2}(L)B_{2}\right],$$ (66)

$$\xi^{t}XK_{Y}\left[D_{2}(L)A_{2}-D_{2}(-L)B_{2}\right]=\xi^{t}K_{R}\left[D_{1}(0)A_{3}-D_{1}(0)B_{3}\right].$$ (67)

Projecting equations (64)–(67) onto the rigid-duct eigenfunctions $\left\{\xi_{m}\right\}$ and using the orthonormality relation (15), the system reduces to


$$A_{1}+B_{1}=X\left[D_{2}(-L)A_{2}+D_{2}(L)B_{2}\right],$$ (68)

$$K_{R}(A_{1}-B_{1})=XK_{Y}\left[D_{2}(-L)A_{2}-D_{2}(L)B_{2}\right],$$ (69)

$$A_{3}+B_{3}=X\left[D_{2}(L)A_{2}+D_{2}(-L)B_{2}\right],$$ (70)

$$K_{R}(A_{3}-B_{3})=XK_{Y}\left[D_{2}(L)A_{2}-D_{2}(-L)B_{2}\right].$$ (71)

Here, *K*_*R*_ and *K*_*Y*_ are diagonal matrices containing the axial wavenumbers in the rigid and lined sections, respectively:


$$K_{R}=diag(s_{0},s_{1},s_{2},\ldots ),\;K_{Y}=diag(\eta_{0},\eta_{1},\eta_{2},\ldots ).$$

Assuming the outlet contains only outgoing waves, we impose ***B***_3_ = **0**. Comparing equations (70) and (71), we solve for ***B***_2_ as:


$$B_{2}=-F^{-1}D_{2}^{-1}(-L)GD_{2}(L)A_{2},$$ (72)

where the matrices *F* and *G* are defined by:


$$F=X+K_{R}^{-1}XK_{Y},\;G=X-K_{R}^{-1}XK_{Y}.$$

Substituting equation (72) into equations (70) and (68) allows us to express ***A***_3_ and ***A***_1_ as:


$$2A_{3}=\left(FD_{2}(L)-F^{-1}D_{2}^{-1}(-L)GD_{2}(L)GD_{2}(-L)\right)A_{2},$$ (73)

$$2A_{1}=\left(FD_{2}(-L)-F^{-1}D_{2}^{-1}(-L)GD_{2}(L)GD_{2}(L)\right)A_{2}.$$ (74)

Similarly, subtracting equation (69) from (68) yields:


$$2B_{1}=\left(GD_{2}(-L)-F^{-1}D_{2}^{-1}(-L)GD_{2}(L)FD_{2}(L)\right)A_{2}.$$ (75)

According to [6], the reflection and transmission coefficients are defined by:


$$T(t)=\frac{A_{3}}{A_{1}},$$ (76)

$$R(r)=\frac{B_{1}}{A_{1}}.$$ (77)

Substituting the expressions for ***A***_3_, ***A***_1_, and ***B***_1_ leads to:


$$\begin{array}{c} T(t)=\left(FD_{2}(L)-F^{-1}D_{2}^{-1}(-L)GD_{2}(L)GD_{2}(-L)\right)\\ \cdot {\left(FD_{2}(-L)-F^{-1}D_{2}^{-1}(-L)GD_{2}(L)GD_{2}(L)\right)}^{-1}, \end{array}$$ (78)

$$\begin{array}{c} R(r)=\left(GD_{2}(-L)-F^{-1}D_{2}^{-1}(-L)GD_{2}(L)FD_{2}(L)\right)\\ \cdot {\left(FD_{2}(-L)-F^{-1}D_{2}^{-1}(-L)GD_{2}(L)GD_{2}(L)\right)}^{-1}. \end{array}$$ (79)

### 3.2 Scattering through double lined cavities

This section addresses the configuration involving double cavities with acoustic liners, as illustrated in Fig 2b. The problem is nondimensionalized following the procedure described in Section 1. The dimensionless acoustic pressure in each region of the duct is defined as:


$$\begin{array}{c} p(x,y)=\left\{ \begin{array}{cc} p_{1}(x,y), & x\leq -2L,\;0\leq y\leq a,\\ p_{2}(x,y), & -2L\leq x\leq -L,\;0\leq y\leq b,\\ p_{3}(x,y), & -L\leq x\leq L,\;0\leq y\leq a,\\ p_{4}(x,y), & L\leq x\leq 2L,\;0\leq y\leq b,\\ p_{5}(x,y), & x\geq 2L,\;0\leq y\leq a. \end{array}\right. \end{array}$$ (80)

The corresponding boundary conditions in dimensional form are specified as follows:

For Region 1 (rigid-walled duct):


$$\frac{\partial p_{1}}{\partial y}=0,\;y=0,\;a,\;x\leq -2L,$$ (81)

For Region 2 (lined upper wall):


$$\frac{\partial p_{2}}{\partial y}=0,\;y=0,\;-2L\leq x\leq -L,$$ (82)

$$\frac{\partial p_{2}}{\partial y}=i\omega Y(\omega )p_{2}(y),\;y=a,\;-2L\leq x\leq -L,$$ (83)

For Region 3 (rigid-walled duct):


$$\frac{\partial p_{3}}{\partial y}=0,\;y=0,\;a,\;-L\leq x\leq L,$$ (84)

For Region 4 (lined upper wall):


$$\frac{\partial p_{4}}{\partial y}=0,\;y=0,\;L\leq x\leq 2L,$$ (85)

$$\frac{\partial p_{4}}{\partial y}=i\omega Y(\omega )p_{4}(y),\;y=a,\;L\leq x\leq 2L,$$ (86)

For Region 5 (rigid-walled duct):


$$\frac{\partial p_{5}}{\partial y}=0,\;y=0,\;a,\;x\geq 2L.$$ (87)

In the next section, a multimodal approach is applied to derive the solution for the acoustic pressure field corresponding to this configuration.

#### 3.2.1 Multimodal solution.

To obtain the multimodal solution, the pressure fields in the duct regions are expressed as linear combinations of transverse modes. These transverse modes and their associated eigenvalues differ based on the eigenvalue problems specific to each region. However, the eigenvalue problems encountered here are identical to those discussed in Section 2.0, and thus, the same modal formulations apply. The pressure in each region can be represented as an eigenfunction expansion:


$$p_{1}(x,y)=\sum_{n=0}^{\infty }A_{1n}\psi_{n}(y)e^{is_{n}(x+2L)}+\sum_{n=0}^{\infty }B_{1n}\psi_{n}(y)e^{-is_{n}(x+2L)},$$ (88)

$$p_{2}(x,y)=\sum_{n=0}^{\infty }A_{2n}\psi_{n}(y)e^{i\eta_{n}(x+L)}+\sum_{n=0}^{\infty }B_{2n}\psi_{n}(y)e^{-i\eta_{n}(x+L)},$$ (89)

$$p_{3}(x,y)=\sum_{n=0}^{\infty }A_{3n}\psi_{n}(y)e^{is_{n}x}+\sum_{n=0}^{\infty }B_{3n}\psi_{n}(y)e^{-is_{n}x},$$ (90)

$$p_{4}(x,y)=\sum_{n=0}^{\infty }A_{4n}\psi_{n}(y)e^{i\eta_{n}(x-L)}+\sum_{n=0}^{\infty }B_{4n}\psi_{n}(y)e^{-i\eta_{n}(x-L)},$$ (91)

$$p_{5}(x,y)=\sum_{n=0}^{\infty }A_{5n}\psi_{n}(y)e^{is_{n}(x-2L)}+\sum_{n=0}^{\infty }B_{5n}\psi_{n}(y)e^{-is_{n}(x-2L)}.$$ (92)

These expressions can be written in matrix form as:


$$p_{1}=\xi^{t}[D_{1}(x+2L)A_{1}+D_{1}(-x-2L)B_{1}],$$ (93)

$$p_{2}=\xi^{t}X[D_{2}(x+L)A_{2}+D_{2}(-x-L)B_{2}],$$ (94)

$$p_{3}=\xi^{t}[D_{1}(x)A_{3}+D_{1}(-x)B_{3}],$$ (95)

$$p_{4}=\xi^{t}X[D_{2}(x-L)A_{4}+D_{2}(-x+L)B_{4}],$$ (96)

$$p_{5}=\xi^{t}[D_{1}(x-2L)A_{5}+D_{1}(-x+2L)B_{5}].$$ (97)

Here, the coefficient vectors


$$\left\{A_{1},A_{2},A_{3},A_{4},A_{5}\right\}\;\text{and}\;\left\{B_{1},B_{2},B_{3},B_{4},B_{5}\right\}$$

represent the unknown modal amplitudes. To facilitate the solution, we assume ***B***_5_ = 0. The remaining unknowns are then determined by enforcing continuity conditions for pressure and normal velocity at the interfaces located at *x* = −2*L*, *x* = −*L*, *x* = *L*, and *x* = 2*L*. The continuity of pressure across these interfaces yields the following matching conditions:


$$p_{1}(-2L,y)=p_{2}(-2L,y),\;0\leq y\leq b,$$ (98)

$$p_{2}(-L,y)=p_{3}(-L,y),\;0\leq y\leq b,$$ (99)

$$p_{3}(L,y)=p_{4}(L,y),\;0\leq y\leq b,$$ (100)

$$p_{4}(2L,y)=p_{5}(2L,y),\;0\leq y\leq b.$$ (101)

respectively. The continuity of normal velocities at the interfaces can be expressed as:


$$\frac{\partial p_{1}}{\partial x}(-2L,y)=\frac{\partial p_{2}}{\partial x}(-2L,y),\;0\leq y\leq b,$$ (102)

$$\frac{\partial p_{2}}{\partial x}(-L,y)=\frac{\partial p_{3}}{\partial x}(-L,y),\;0\leq y\leq b,$$ (103)

$$\frac{\partial p_{3}}{\partial x}(L,y)=\frac{\partial p_{4}}{\partial x}(L,y),\;0\leq y\leq b,$$ (104)

$$\frac{\partial p_{4}}{\partial x}(2L,y)=\frac{\partial p_{5}}{\partial x}(2L,y),\;0\leq y\leq b.$$ (105)

Substituting equations (93)–(97) into the continuity conditions (98)–(105), we obtain the following interface conditions:


$$\xi^{t}\left[D_{1}(0)A_{1}+D_{1}(0)B_{1}\right]=\xi^{t}X\left[D_{2}(-L)A_{2}+D_{2}(L)B_{2}\right],$$ (106)

$$\xi^{t}X\left[D_{2}(0)A_{2}+D_{2}(0)B_{2}\right]=\xi^{t}\left[D_{1}(-L)A_{3}+D_{1}(L)B_{3}\right],$$ (107)

$$\xi^{t}\left[D_{1}(L)A_{3}+D_{1}(-L)B_{3}\right]=\xi^{t}X\left[D_{2}(0)A_{4}+D_{2}(0)B_{4}\right],$$ (108)

$$\xi^{t}X\left[D_{2}(L)A_{4}+D_{2}(-L)B_{4}\right]=\xi^{t}\left[D_{1}(0)A_{5}+D_{1}(0)B_{5}\right],$$ (109)

$$\xi^{t}K_{R}\left[D_{1}(0)A_{1}-D_{1}(0)B_{1}\right]=\xi^{t}XK_{Y}\left[D_{2}(-L)A_{2}-D_{2}(L)B_{2}\right],$$ (110)

$$\xi^{t}XK_{Y}\left[D_{2}(0)A_{2}-D_{2}(0)B_{2}\right]=\xi^{t}K_{R}\left[D_{1}(-L)A_{3}-D_{1}(L)B_{3}\right],$$ (111)

$$\xi^{t}K_{R}\left[D_{1}(L)A_{3}-D_{1}(-L)B_{3}\right]=\xi^{t}XK_{Y}\left[D_{2}(0)A_{4}-D_{2}(0)B_{4}\right],$$ (112)

$$\xi^{t}XK_{Y}\left[D_{2}(L)A_{4}-D_{2}(-L)B_{4}\right]=\xi^{t}K_{R}\left[D_{1}(0)A_{5}-D_{1}(0)B_{5}\right].$$ (113)

Projecting equations (106)–(113) onto the rigid-duct eigenfunctions $\left\{\xi_{m}\right\}$ and using the orthonormality relation (15), the system reduces to


$$A_{1}+B_{1}=X\left(D_{2}(-L)A_{2}+D_{2}(L)B_{2}\right),$$ (114)

$$K_{R}(A_{1}-B_{1})=XK_{Y}\left(D_{2}(-L)A_{2}-D_{2}(L)B_{2}\right),$$ (115)

$$X(A_{2}+B_{2})=D_{1}(-L)A_{3}+D_{1}(L)B_{3},$$ (116)

$$XK_{Y}(A_{2}-B_{2})=K_{R}\left(D_{1}(-L)A_{3}-D_{1}(L)B_{3}\right),$$ (117)

$$X(A_{4}+B_{4})=D_{1}(L)A_{3}+D_{1}(-L)B_{3},$$ (118)

$$XK_{Y}(A_{4}-B_{4})=K_{R}\left(D_{1}(L)A_{3}-D_{1}(-L)B_{3}\right),$$ (119)

$$A_{5}+B_{5}=X\left(D_{2}(L)A_{4}+D_{2}(-L)B_{4}\right),$$ (120)

$$K_{R}(A_{5}-B_{5})=XK_{Y}\left(D_{2}(L)A_{4}-D_{2}(-L)B_{4}\right).$$ (121)

Assuming ***B***_5_ = 0 and comparing equations (120) and (121), we can directly solve for ***B***_4_ as:


$$B_{4}=-F_{1}^{-1}D_{2}^{-1}(-L)G_{1}D_{2}(L)A_{4},$$ (122)

where


$$F_{1}=X+K_{R}^{-1}XK_{Y},\;G_{1}=X-K_{R}^{-1}XK_{Y}.$$

Adding equations (118) and (119), multiplying by $D_{1}^{-1}(L)$, and substituting $B_{4}$ yields:


$$2A_{3}=\left(F_{1}D_{1}^{-1}(L)-G_{1}D_{1}^{-1}(L)F_{1}^{-1}D_{2}^{-1}(-L)G_{1}D_{2}(L)\right)A_{4}.$$ (123)

Similarly, subtracting equations (118) and (119), multiplying by $D_{1}^{-1}(-L)$, and using the value of $B_{4}$, we get:


$$2B_{3}=\left(G_{1}D_{1}^{-1}(-L)-F_{1}D_{1}^{-1}(-L)F_{1}^{-1}D_{2}^{-1}(-L)G_{1}D_{2}(L)\right)A_{4}.$$ (124)

Next, adding equations (116) and (117), multiplying by 2, and substituting expressions for $2A_{3}$ and $2B_{3}$, we obtain:


$$4A_{2}=\left(F_{2}D_{1}(-L)E_{1}+G_{2}D_{1}(L)E_{2}\right)A_{4},$$ (125)

where


$$F_{2}=X^{-1}+K_{Y}^{-1}X^{-1}K_{R},\;G_{2}=X^{-1}-K_{Y}^{-1}X^{-1}K_{R},$$

$$E_{1}=F_{1}D_{1}^{-1}(L)-G_{1}D_{1}^{-1}(L)F_{1}^{-1}D_{2}^{-1}(-L)G_{1}D_{2}(L),$$

$$E_{2}=G_{1}D_{1}^{-1}(-L)-F_{1}D_{1}^{-1}(-L)F_{1}^{-1}D_{2}^{-1}(-L)G_{1}D_{2}(L).$$

Subtracting equations (116) and (117), and again using the values of $2A_{3}$ and $2B_{3}$, we find:


$$4B_{2}=\left(G_{2}D_{1}(-L)E_{1}+F_{2}D_{1}(L)E_{2}\right)A_{4}.$$ (126)

Now, adding equations (114) and (115), multiplying by 4, and substituting $4A_{2}$ and $4B_{2}$, we get:


$$8A_{1}=\left(F_{1}D_{2}(-L)H_{1}+G_{1}D_{2}(L)H_{2}\right)A_{4},$$ (127)

and subtracting the same equations leads to:


$$8B_{1}=\left(G_{1}D_{2}(-L)H_{1}+F_{1}D_{2}(L)H_{2}\right)A_{4},$$ (128)

where


$$H_{1}=F_{2}D_{1}(-L)E_{1}+G_{2}D_{1}(L)E_{2},\;H_{2}=G_{2}D_{1}(-L)E_{1}+F_{2}D_{1}(L)E_{2}.$$

Adding equations (120) and (121), and substituting the value of $B_{4}$, we finally obtain:


$$2A_{5}=\left(F_{1}D_{2}(L)-G_{1}D_{2}(-L)F_{1}^{-1}D_{2}^{-1}(-L)G_{1}D_{2}(L)\right)A_{4}.$$ (129)

According to [6], the transmission and reflection coefficients are given by:


$$T(t)=\frac{A_{5}}{A_{1}},$$ (130)

$$R(r)=\frac{B_{1}}{A_{1}}.$$ (131)

Substituting the expressions for ***A***_5_ and ***A***_1_, the transmission coefficient becomes:


$$\begin{array}{c} T(t)=\left(F_{1}D_{2}(L)-G_{1}D_{2}(-L)F_{1}^{-1}D_{2}^{-1}(-L)G_{1}D_{2}(L)\right)\\ \cdot {\left(F_{1}D_{2}(-L)H_{1}+G_{1}D_{2}(L)H_{2}\right)}^{-1}. \end{array}$$ (132)

Similarly, the reflection coefficient is given by:


$$\begin{array}{c} R(r)=\left(G_{1}D_{2}(-L)H_{1}+F_{1}D_{2}(L)H_{2}\right)\\ \cdot {\left(F_{1}D_{2}(-L)H_{1}+G_{1}D_{2}(L)H_{2}\right)}^{-1}. \end{array}$$ (133)

## 4 Numerical results

### 4.1 Scattering amplitudes in rigid and lined duct

In this section, numerical results are presented. The model problem involves excitation by a plane piston oscillating with a constant velocity *U*. The duct is partially lined with a reactive acoustic lining in the region a≤y≤b. For the computational analysis, the dimensionless geometric parameters are taken as *H* = 3, $a=\frac{25}{3}$, and $b=\frac{30}{3}$. The speed of sound is assumed to be 343.5 m/s.

#### 4.1.1 Scattering amplitudes in rigid duct.

**Fig 3:**
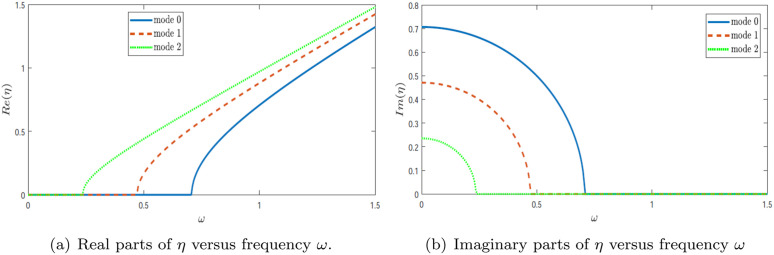
Trajectories of the real parts and imaginary parts of η versus frequency ω.

**Fig 4:**
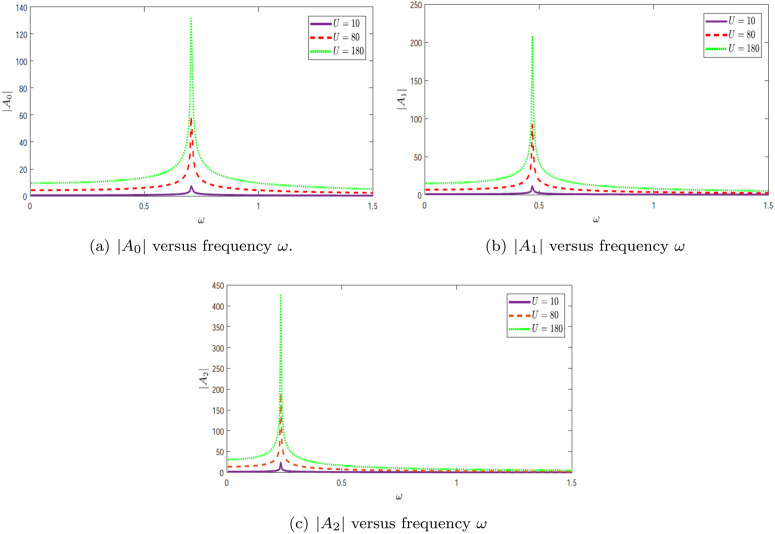
The absolute value of fundamental mode amplitude $|A_{n}|,\;n=0,1,2$ versus frequency ω.

In Fig 3, the variation of the wavenumber for modes 0, 1, and 2 is plotted as a function of frequency ω. Here, these branches correspond to the first three eigenmodes of the *lined* region (Region 2), i.e., to the axial wavenumbers $\eta_{n}(\omega )$ obtained from the dispersion relation in Section 2.2. The real and imaginary components of the modal wavenumber η are separately illustrated in Figs 3(a) and 3(b), respectively. These figures capture the evolution of modal behavior with frequency and highlight the influence of mode trajectories on wave propagation, as reflected by their respective absolute amplitudes. Fig 3(a) shows the real part ℜ(η), which characterizes the axial propagation of each mode. At lower frequencies, the lined-region eigenbranches shown here are cut off and have vanishing *real* part of the axial wavenumber, while their *imaginary* parts remain non-zero so that the lined modes are evanescent in the axial direction. As frequency increases, each mode becomes propagating beyond its specific cutoff frequency, where ℜ(η) begins to rise. The fundamental mode (mode 0) transitions first, followed by mode 1 and then mode 2, with each higher-order mode requiring a higher threshold frequency. After becoming propagative, the real part of the wavenumber increases monotonically with frequency, indicating increasing phase propagation along the duct. Fig 3(b) presents the imaginary part ℑ(η), which quantifies attenuation. For frequencies below the cutoff, the imaginary component is non-zero, signifying evanescent decay in the axial direction. As frequency approaches the cutoff, this imaginary component decreases and eventually vanishes, indicating the onset of propagation. The attenuation is most significant for the lower-order modes at low frequencies and becomes negligible once the mode is propagating. Together, these figures clearly depict the modal dispersion characteristics and cutoff phenomena typical in waveguide acoustics. The frequencies at which ℜ(η) emerges and ℑ(η) disappears define the modal cutoffs—essential for predicting how many modes are actively propagating at any given frequency within the duct. In Fig 4, the absolute value of the fundamental amplitude is plotted as a function of frequency ω for various piston velocities *U*. The results show that the amplitude magnitude varies significantly with changes in piston velocity. Fig 4(a) presents the amplitude |*A*_0_| versus frequency ω for three interaction strengths: *U* = 10, *U* = 80, and *U* = 180. A distinct resonance peak appears at low frequencies, particularly for *U* = 80 and *U* = 180. As *U* increases, the peak becomes markedly higher, indicating that stronger interaction enhances the system’s resonant response at low frequencies. In Fig 4(b), the amplitude |*A*_1_| is plotted against frequency ω. Similar to the fundamental mode, a prominent peak is observed, which shifts slightly toward higher frequencies with increasing *U*. The peak also becomes narrower and more pronounced, suggesting that increased interaction strength modifies the resonance characteristics of the first higher mode. Fig 4(c) shows the amplitude |*A*_2_| as a function of frequency ω. A substantial rise in amplitude is evident, especially for *U* = 180, where the peak is sharp and significantly elevated. This indicates that higher modes are more sensitive to interaction strength, marking a transition to a regime dominated by nonlinear interaction effects. The pronounced peak at higher values of *U* highlights the system’s complex dynamic behavior under strong coupling. Overall, these figures demonstrate how the interaction strength *U* critically influences resonant behavior across different modes, with higher modes exhibiting increased sensitivity and nonlinear response.

#### 4.1.2 Scattering amplitudes in lined duct.

**Fig 5:**
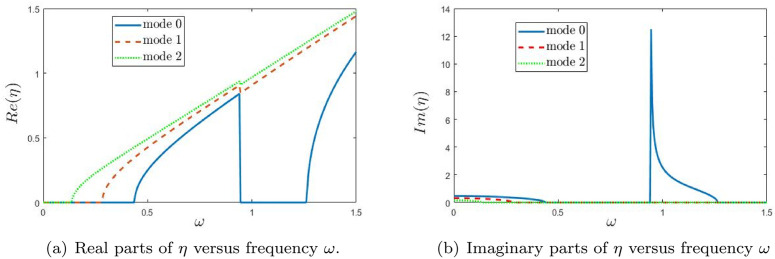
Trajectories of the real parts and imaginary parts of η versus frequency ω.

**Fig 6:**
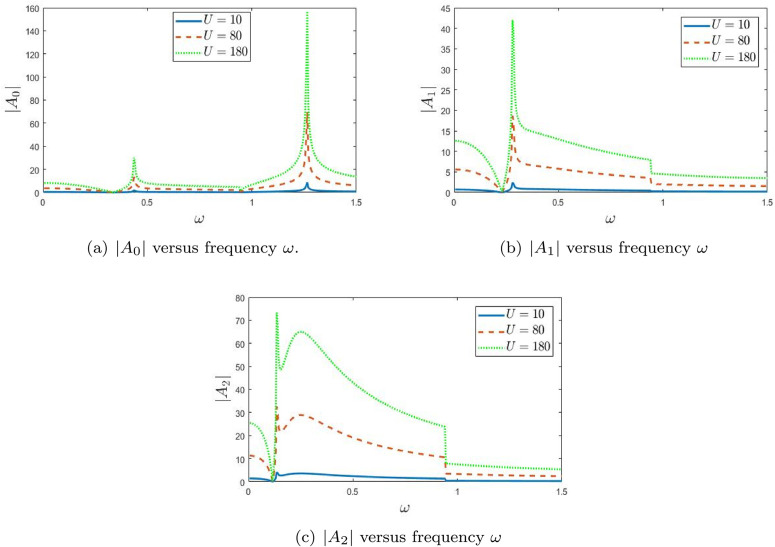
The absolute value of fundamental mode amplitude $|A_{n}|,\;n=0,1,2$ versus frequency ω.

In Fig 3, the real and imaginary parts of the wavenumber η are plotted against frequency ω. These trajectories significantly influence wave propagation, and their impact is reflected in the absolute amplitude plots shown in the corresponding figures. The absolute value of the fundamental amplitude is displayed as a function of frequency for various piston velocities *U*. It is evident that changing the piston velocity alters the amplitude magnitude. Notably, the sharp peaks observed in the curves across all figures are attributed to variations in eigenvalues. This is further substantiated by the trajectories of the modal wavenumber η. Fig 5(a) presents the real part of η as a function of frequency ω for three distinct modes: mode 0, mode 1, and mode 2. The results reveal mode-dependent behaviors. For mode 0, the real part Re(η) increases smoothly with frequency, indicating stable propagation characteristics. Mode 1, however, exhibits abrupt transitions, suggesting possible phase shifts or dynamic instabilities at specific frequencies. These sharp transitions indicate critical frequencies where the system undergoes notable changes in behavior. Mode 2, shown as a dotted line, demonstrates a nonlinear trend, implying a more intricate interaction between frequency and the system parameters. Fig 5(b) shows the imaginary part of η as a function of frequency ω, complementing the trends seen in Fig 3(a). For mode 0, the imaginary part remains consistently low, indicating weak attenuation and stable energy transmission. In contrast, mode 1 features a sharp peak at a particular frequency, signifying a resonant response and potential for strong energy absorption. This highlights the system’s sensitivity to certain frequencies. Mode 2, by comparison, displays a relatively small and less variable imaginary component, indicating a weaker dependence on frequency. Together, these figures offer meaningful insights into the modal dynamics of the system. The contrasting behaviors in the real and imaginary parts of η underscore the complex frequency-dependent nature of wave propagation. The presence of sharp transitions and resonant peaks reveals that the system is particularly responsive near specific frequencies, likely associated with eigenvalue interactions and modal coupling effects. In Fig 6, the absolute values of the fundamental mode amplitudes |*A*_0_|, |*A*_1_|, and |*A*_2_| are presented. These modes are generated by a plane piston oscillating with velocities *U* = 10 m/s, 80 m/s, and 180 m/s. It is evident from the figures that the amplitudes |*A*_0_|, |*A*_1_|, and |*A*_2_| increase as the piston velocity *U* is increased, indicating a strong dependence of amplitude response on the driving velocity. Fig 6(a) displays the amplitude |*A*_0_| as a function of frequency ω for the three piston velocities. A pronounced peak appears at low frequencies, especially for *U* = 80 and *U* = 180, indicating strong resonance behavior. As *U* increases, the height of the peak grows significantly, suggesting that greater interaction strength enhances the amplitude at resonance. This trend reflects the system’s high sensitivity to changes in *U*, particularly in the low-frequency range. In Fig 6, the absolute values of the fundamental mode amplitudes |*A*_0_|, |*A*_1_|, and |*A*_2_| are presented. These modes are generated by a plane piston oscillating with velocities *U* = 10 m/s, 80 m/s, and 180 m/s. It is evident from the figures that the amplitudes |*A*_0_|, |*A*_1_|, and |*A*_2_| increase as the piston velocity *U* is increased, indicating a strong dependence of amplitude response on the driving velocity. This demonstrates that stronger forcing enhances the excitation of the duct modes, particularly near resonance frequencies.

Fig 6(a) displays the amplitude |*A*_0_| as a function of frequency ω for the three piston velocities. A pronounced peak appears at low frequencies, especially for *U* = 80 and *U* = 180, indicating strong resonance behavior. As *U* increases, the height of the peak grows significantly, suggesting that greater interaction strength enhances the amplitude at resonance. This trend reflects the system’s high sensitivity to changes in *U*, particularly in the low-frequency range. Thus, the dominant low-frequency response of the fundamental mode is governed mainly by cavity-resonance effects, which become more pronounced under stronger excitation.

Fig 6(b) presents the amplitude |*A*_1_| plotted against frequency ω. A prominent resonance peak is again observed, though it shifts slightly toward higher frequencies with increasing *U*. Interestingly, the overall amplitude response becomes more intricate for higher piston velocities, with a reduction in peak magnitude at the largest value of *U*. This implies that the interaction strength modifies the resonance behavior of this mode, suggesting more complex dynamic behavior as *U* increases. The shift and redistribution of the peaks indicate that the first higher mode is more strongly affected by modal interaction than the fundamental mode.

In Fig 6(c), the amplitude |*A*_2_| is shown as a function of frequency ω. Compared to the previous modes, the amplitude displays a more subdued resonance peak, and it generally decreases with increasing frequency. For *U* = 80 and *U* = 180, a gradual decline in amplitude is observed, indicating that higher-order modes are less responsive to frequency variations. This suggests a shift in the system’s dynamics, where higher modes become less dominant as interaction strength increases. This behavior indicates that the second higher mode contributes less to the overall response except near isolated resonance regions.

Taken together, these figures highlight the influence of piston velocity *U* on the amplitude responses of different modes. While lower modes exhibit enhanced resonance with increased interaction strength, higher modes show more complex and diminished responses. Overall, Fig 6 shows that the main effect of increasing *U* is not only amplitude growth, but also a clearer separation between strongly resonant low-order modes and weaker higher-order modal responses. This behavior points to the presence of nonlinear effects in the system that become more pronounced with increasing *U*, resulting in intricate wave dynamics.

### 4.2 Scattering amplitudes against frequency with single and double lined chamber

In this section, the numerical results are presented. The system is excited by the fundamental duct mode in the inlet region, which undergoes scattering upon interaction with the lined chamber. The absolute amplitudes of the first three reflected modes in the inlet, as well as the corresponding transmitted modes in the outlet, are plotted as functions of frequency. These modes are responsible for carrying the majority of the incident energy. The primary objective is to examine how the scattering fields vary with frequency and how these variations are influenced by the presence of the lined chamber. For all numerical simulations, the speed of sound is taken as *c* = 343.5 m/s, while the dimensional heights are fixed at *a* = 25/6 and *b* = 30/6.

**Fig 7:**
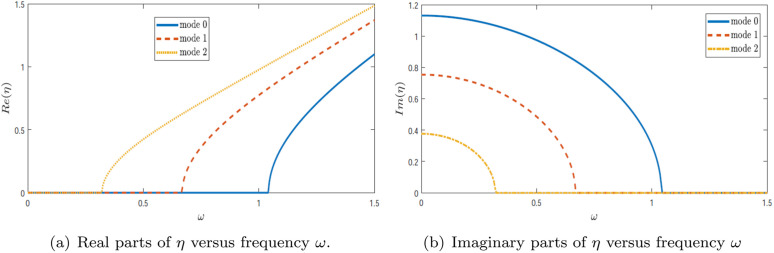
Trajectories of the real parts and imaginary parts of η versus frequency ω.

Fig 7 displays the real and imaginary components of the wavenumber η as functions of frequency ω over the range 0≤ω≤1.5, for a configuration with a single-lined chamber. For numerical computations, the speed of sound is fixed at *c* = 343.5 m/s, and the dimensional heights are taken as *a* = 25/6 and *b* = 30/6. These figures also depict the absolute values of the fundamental reflected and transmitted mode amplitudes. From the plots, it is evident that significant spikes occur in the reflected amplitudes within the range 0.3≤ω≤0.6, and in the transmitted amplitudes within 0.6≤ω≤0.8. These spikes are attributed to variations in the system’s eigenvalues, indicating resonant behavior or mode conversion phenomena. Hence, Fig 7 provides the spectral explanation for the peaks observed later in the scattering amplitudes.

Fig 7(a) shows the real part of η as a function of frequency ω for three distinct modes: mode 0, mode 1, and mode 2. The plot reveals clear differences in modal behavior. Mode 0 exhibits a smooth and continuous increase in Re(η), suggesting stable and predictable propagation characteristics. In contrast, mode 1 shows a more complex trend, characterized by a sharp rise followed by a region of stabilization, which may indicate a phase transition or critical dynamic shift. Mode 2 presents a non-linear increase in Re(η), diverging from the trends seen in the lower modes, possibly reflecting a different underlying physical mechanism. These changes in Re(η) indicate how the axial propagation characteristics evolve with frequency and help identify the frequency ranges where stronger modal interaction is expected.

Fig 7(b) presents the imaginary part of η over the same frequency range. For mode 0, the imaginary component remains small and nearly constant, indicating negligible energy dissipation. Mode 1 exhibits a gradual decline in the imaginary part as frequency increases, suggesting moderate damping behavior. Meanwhile, mode 2 shows a more intricate response—initially undergoing a steep drop followed by gradual stabilization—implying more complex dynamics, possibly linked to coupling or modal interference. In particular, reductions in Im(η) are associated with weaker evanescence and the onset of stronger participation of the corresponding mode in the scattering process.

**Fig 8:**
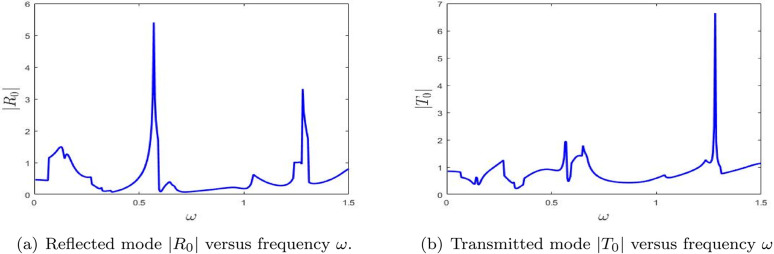
The absolute fundamental reflected mode |*R*_0_| and transmitted mode |*T*_0_| versus frequency ω.

**Fig 9:**
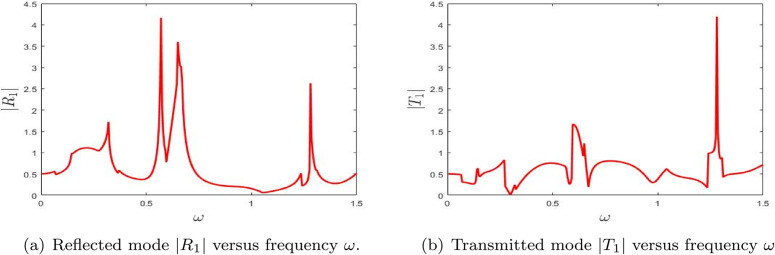
The absolute fundamental reflected mode |*R*_1_| and transmitted mode |*T*_1_| versus frequency ω.

**Fig 10:**
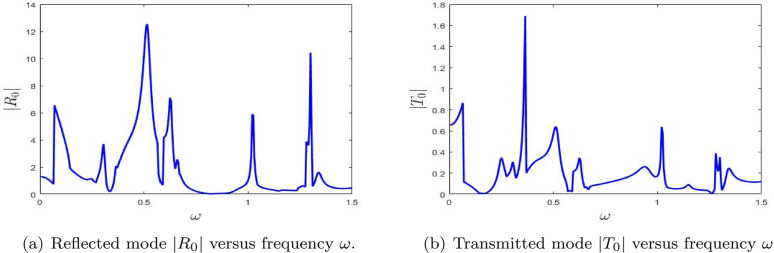
The absolute fundamental reflected mode |*R*_0_| and transmitted mode |*T*_0_| versus frequency ω.

**Fig 11:**
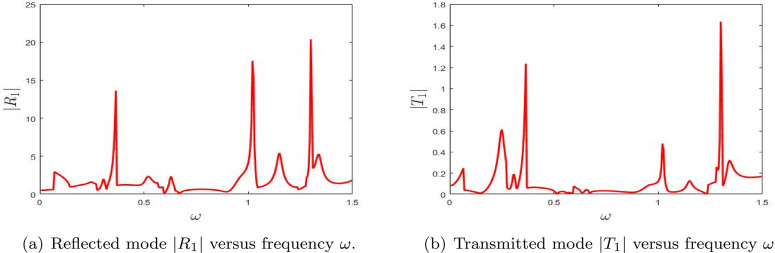
The absolute fundamental reflected mode |*R*_1_| and transmitted mode |*T*_1_| versus frequency ω.

**Fig 12:**
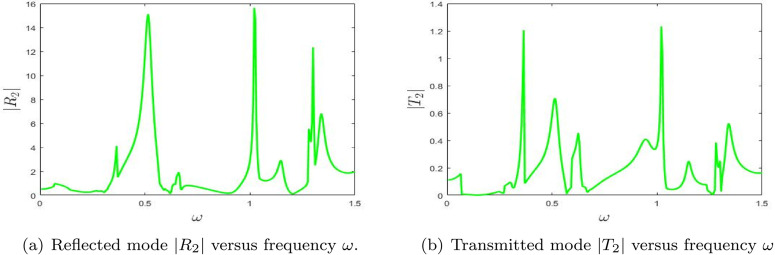
The absolute fundamental reflected mode |*R*_2_| and transmitted mode |*T*_2_| versus frequency ω.

Collectively, these figures reveal the nuanced interplay between frequency and wave propagation characteristics across different modes. The contrasting behaviors of the real and imaginary components of η emphasize the system’s sensitivity to frequency, especially in mode 1, where transitional behaviors are most apparent. While mode 0 reflects stable dynamics, the higher modes, particularly modes 1 and 2, exhibit increasingly complex and nonlinear responses, indicating the influence of more sophisticated interaction mechanisms. Therefore, the eigenvalue trajectories provide a direct physical basis for interpreting the resonance peaks observed in Figs 8–12.

#### 4.2.1 Scattering amplitudes against frequency with single lined chamber.

Fig 8 displays the reflected and transmitted amplitudes as functions of frequency over the range 0≤ω≤1.5 for a configuration with a single lined chamber. For all numerical computations, the speed of sound is set to *c* = 343.5 m/s, with fixed dimensional heights *a* = 25/6 and *b* = 30/6. Fig 8(a) and 8(b) respectively illustrate the absolute values of the fundamental reflected amplitude |*R*_0_| and the fundamental transmitted amplitude |*T*_0_|. A prominent spike is observed in the reflected amplitude within the frequency range 0.3≤ω≤0.6, while a similar spike in the transmitted amplitude appears in the interval 0.6≤ω≤0.8. These spikes are attributed to variations in the system’s eigenvalues, indicating the presence of resonant effects. This shows that the single lined chamber acts selectively, with different frequency windows favoring reflection and transmission.

Fig 8(a) presents the reflectance |*R*_0_| as a function of frequency ω. A distinct peak occurs around ω=0.5, signifying a strong reflective response, likely due to resonance within the lined chamber. Additional smaller peaks and oscillations suggest the occurrence of multiple resonant or interference effects across the frequency spectrum. These features indicate that the system exhibits complex reflective behavior that is highly sensitive to changes in frequency. Thus, the main lesson from Fig 8(a) is that the chamber can strongly suppress forward propagation near selected resonant frequencies.

Fig 8(b) shows the transmittance |*T*_0_| plotted against frequency ω. The transmittance curve also reveals multiple peaks, including a significant spike near the frequency where reflectance is maximized. This implies a close interplay between reflection and transmission phenomena, which is typical in systems governed by resonant and interference mechanisms. The fluctuations observed in |*T*_0_| suggest that the system undergoes frequency-dependent variations in energy transmission, further reinforcing the resonance behavior noted in the reflectance data. The transmitted response therefore confirms that resonance does not simply enhance reflection, but redistributes the acoustic energy in a frequency-dependent manner.

Collectively, these figures provide key insights into the frequency-dependent reflective and transmissive properties of the system. The sharp peaks observed in both |*R*_0_| and |*T*_0_| emphasize the system’s high sensitivity to specific frequencies. The correlation between the peaks in reflectance and transmittance suggests that underlying mechanisms—such as resonance and modal interference—play a significant role in shaping the wave behavior within the lined chamber. Overall, Fig 8 shows that even the fundamental scattered mode already captures the main resonant signature of the single-chamber configuration.

It is observed that, initially, the reflected amplitude remains low but increases with frequency, reaching its maximum within the range 0.5≤ω≤0.7. In contrast, the transmitted amplitude exhibits prominent spikes in the interval 0.6≤ω≤1.3, indicating a distinct frequency-dependent behavior of the system. This separation between reflection-dominated and transmission-enhanced ranges further illustrates the filtering action of the lined chamber.

Fig 9 illustrates the frequency-dependent behavior of the reflected and transmitted amplitudes for the first higher mode, denoted by |*R*_1_| and |*T*_1_|, respectively. Fig 9(a) presents the reflectance |*R*_1_| as a function of frequency ω. Similar to the trends seen in transmittance, the reflectance curve exhibits multiple pronounced peaks, particularly near the frequencies where transmittance also reaches high values. These peaks indicate that, at certain resonant frequencies, a significant portion of the incident energy is reflected. The intricate structure of the |*R*_1_| curve suggests the presence of multiple resonances and interference phenomena, highlighting the system’s complex and frequency-sensitive reflective behavior. Compared with the fundamental mode, the more oscillatory structure of |*R*_1_| indicates stronger sensitivity of the first higher mode to modal conversion.

Fig 9(b) shows the transmittance |*T*_1_| as a function of frequency ω. Notable peaks are observed, especially around ω=0.5 and ω=1.4, corresponding to frequencies where the system permits enhanced transmission. These spikes signify resonance conditions that facilitate strong energy transfer through the chamber. The overall variation in transmittance across the frequency range indicates that the system is highly sensitive to frequency changes, likely due to modal interactions and interference effects. Hence, Fig 9(b) shows that the first higher mode becomes important only in selected frequency bands where coupling with the chamber is strongest.

Together, these figures underscore the strong interdependence between transmittance and reflectance in the system. The alignment of peaks in |*T*_1_| and |*R*_1_| points to a resonant behavior wherein energy is either reflected or transmitted depending on the frequency. The observed fluctuations in both amplitudes reflect the complex nature of wave interaction within the lined chamber, governed by resonance and interference phenomena. The key point is that higher-mode scattering is more localized in frequency than the fundamental response.

#### 4.2.2 Scattering amplitudes aginst frequency with double lined chamber.

Fig 10 displays the absolute values of the scattering amplitudes for a configuration with a double lined chamber. The numerical parameters used remain consistent with those employed for the single lined chamber case. These plots illustrate the absolute values of the fundamental reflected and transmitted mode amplitudes. Compared to the single lined chamber configuration, noticeable fluctuations in the scattering amplitudes are observed. These fluctuations can be attributed to the introduction of the additional lined chamber, which alters the wave interaction dynamics within the system. The additional chamber introduces extra resonance paths and therefore produces a richer scattering structure than in the single-chamber case.

Fig 10(a) shows the reflectance |*R*_0_| as a function of frequency ω. The plot reveals several pronounced peaks, especially near ω=0.5 and extending into higher frequencies up to ω=1.5. These peaks signify strong reflective behavior at certain frequencies, indicative of resonance phenomena where a substantial portion of the incident energy is reflected. The presence of multiple peaks suggests the influence of modal interference and the excitation of several resonant modes within the double chamber configuration. Relative to Fig 8(a), the denser peak pattern shows that adding a second chamber broadens the range of frequencies over which strong reflection can occur.

Fig 10b presents the transmittance |*T*_0_| as a function of frequency ω. While this plot also exhibits distinct peaks, the amplitudes are generally lower than those observed in the reflectance curve. The most prominent transmittance peak appears near ω=0.5, suggesting that, at this frequency, a portion of the energy is transmitted through the system despite significant reflection. The overall fluctuations in the transmittance curve indicate a complex, frequency-dependent response, characteristic of systems exhibiting strong resonant and interference effects. This reduced transmission compared with the single-chamber case indicates that the second lined section strengthens the overall blocking effect.

Together, these figures provide valuable insight into the interplay between reflection and transmission in the presence of a double lined chamber. The alignment of peaks in both |*R*_0_| and |*T*_0_| highlights the role of resonant conditions in shaping the scattering behavior. The enhanced reflectance at specific frequencies underscores the system’s ability to efficiently reflect acoustic energy, while the comparatively lower transmittance suggests that most of the energy is confined or redirected due to the added chamber, reflecting the complex nature of wave propagation in such configurations. Overall, Fig 10 shows that the double-chamber arrangement enhances frequency selectivity and favors stronger reflection over a wider spectral range.

Fig 11 displays the absolute values of the secondary reflected and transmitted mode amplitudes, |*R*_1_| and |*T*_1_|, respectively, for the configuration with a double lined chamber. Compared to the corresponding results from the single lined chamber case, only minor variations in the scattering amplitudes are observed, indicating that the addition of a second chamber introduces subtle changes in the wave interaction behavior. Thus, for the first higher mode, the effect of the second chamber is present but less dramatic than for the fundamental mode.

The analysis of the numerical results provides valuable insight into the frequency-dependent behavior of reflectance and transmittance, as shown in Fig 11(a) and 11(b), respectively. In the reflectance plot (Fig 11(a)), several prominent peaks are evident: at ω≈0.3 with $|R_{1}|\approx 5.0$, ω≈0.6 where $|R_{1}|\approx 20.0$, ω≈1.1 with $|R_{1}|\approx 15.0$, and ω≈1.4 reaching $|R_{1}|\approx 18.0$. These peaks signify strong resonant behavior, indicating that the system supports multiple resonance modes within the analyzed frequency range. The presence of several separated peaks indicates that the first higher mode responds to multiple distinct resonance conditions in the double-chamber geometry.

The transmittance data in Fig 11(b) also highlights several key frequencies: ω≈0.3 with $|T_{1}|\approx 0.2$, ω≈0.6 where $|T_{1}|\approx 1.6$, ω≈1.1 with $|T_{1}|\approx 0.3$, and ω≈1.4 showing $|T_{1}|\approx 0.5$. These values are considerably lower than those observed for reflectance, suggesting that while a portion of the acoustic energy is transmitted, a dominant share is reflected at these frequencies. Hence, the main effect of the double chamber for this mode is to maintain transmission at relatively low levels while preserving strong reflective resonances.

The alignment of peak locations in both reflectance and transmittance curves confirms the presence of complex resonant dynamics governing the energy distribution. These results emphasize the significance of modal interactions and interference effects in shaping the scattering behavior of the system. Such findings are not only relevant for acoustic applications but also offer valuable analogies for wave manipulation in optical and material systems, providing a foundation for further investigations into the underlying physical mechanisms. In summary, Fig 11 shows that the double-chamber effect on the first higher mode is mainly qualitative, through additional resonance structure, rather than through a large overall amplification.

The numerical results for the reflectance |*R*_2_| and transmittance |*T*_2_| as functions of frequency ω, presented in Fig 12, provide valuable insights into the wave propagation behavior of the system. The reflectance plot exhibits several notable peaks, specifically at ω≈0.5 with $|R_{2}|\approx 8.0$, ω≈0.8 with $|R_{2}|\approx 16.0$, ω≈1.0 where $|R_{2}|\approx 12.0$, and ω≈1.4 reaching $|R_{2}|\approx 14.0$. These peaks are indicative of strong resonance effects, where a significant portion of the incident energy is reflected. For the second higher mode, these resonances are again concentrated in isolated frequency bands, confirming the selective nature of higher-mode scattering.

In contrast, the transmittance plot shows corresponding but smaller peaks, such as at ω≈0.5 with $|T_{2}|\approx 0.4$, ω≈0.8 where $|T_{2}|\approx 1.2$, and ω≈1.0 with $|T_{2}|\approx 0.3$. These relatively lower values indicate that transmission is less dominant, with most of the energy being reflected by the system. This means that, in the double-chamber configuration, the second higher mode contributes mainly to the reflective part of the scattering response.

The alignment of the peaks in both |*R*_2_| and |*T*_2_| suggests a strong coupling between reflection and transmission mechanisms, driven by resonance phenomena. This interplay highlights the presence of complex wave interactions and modal dynamics that play a crucial role in the frequency-dependent distribution of energy within the system. Overall, Fig 12 confirms that higher-order responses in the double-lined chamber remain strongly resonance-controlled and are more localized in frequency than the fundamental response. Note that the peaks are linked to eigenvalue trajectories, occurring near cut-on transitions of the modes. Truncation tests confirm that their locations remain stable, indicating they are not numerical artifacts.

### 4.3 Energy balance and solution validation

The analytical solution is expressed in an orthogonal eigenfunction basis, which leads to a non-singular coefficient matrix and rapidly convergent modal expansions. To validate both the algebra and the numerical implementation, we examine the balance between incident, reflected, and transmitted power, thereby checking the internal consistency of the scheme. These power-balance checks provide an analytical and numerical cross-validation of the framework in the absence of dedicated experimental data for the single- and double-cavity geometries considered here. For time-harmonic fields with time dependence $e^{-i\omega t}$, the time-averaged axial acoustic power flux through a duct cross-section is given by


$$P=\frac{1}{2\rho \omega }\int_{0}^{a}ℑ\;(p(x,y)\;\partial_{x}\overset{―}{p(x,y)})\;dy,$$ (134)

where ρ is the fluid density. In the rigid inlet and outlet regions, the pressure field is expanded in duct modes of the form


$$p(x,y)=\sum_{n=0}^{\infty }C_{n}\;\psi_{n}(y)\;e^{i\kappa_{n}x},$$ (135)

where $\psi_{n}$ are orthonormal eigenfunctions and $\kappa_{n}=\pm s_{n}$ denotes the axial wavenumber of mode *n*. Using orthogonality, one obtains


$$P=\frac{1}{2\rho \omega }\sum_{n}ℜ(\kappa_{n})\;|C_{n}{|}^{2}.$$ (136)

Hence the contribution of each propagating mode is proportional to $ℜ(\kappa_{n})\;|C_{n}{|}^{2}$. In the single- and double-cavity configurations, the inlet and outlet fields consist of an incident fundamental mode and scattered reflected and transmitted modes. Denoting by *R*_*n*_ and *T*_*n*_ the reflection and transmission coefficients of mode *n*, normalised with respect to the incident fundamental, the reflected and transmitted power coefficients are


$${𝒫}_{R}(\omega )=\sum_{n\in 𝒫}\frac{ℜ(s_{n})}{ℜ(s_{0})}\;|R_{n}(\omega ){|}^{2},\;{𝒫}_{T}(\omega )=\sum_{n\in 𝒫}\frac{ℜ(s_{n})}{ℜ(s_{0})}\;|T_{n}(\omega ){|}^{2},$$ (137)

where 𝒫 denotes the set of propagating modes ($ℜ(s_{n})>0$). The incident field is the plane-wave mode (*n* = 0) with unit amplitude, and the modal basis is normalized using the rigid-duct orthogonality relations. For the purely reactive liners considered here the system is lossless, and the modal powers satisfy the energy-balance identity


$$1={𝒫}_{R}(\omega )+{𝒫}_{T}(\omega ).$$ (138)

In terms of the transmitted power, the usual transmission loss (in decibels) is defined by


$$\mathrm{TL}(\omega )=-10\log_{10}{𝒫}_{T}(\omega ),$$ (139)

which is the quantity commonly used to characterise muffler and liner performance. In the numerical implementation we evaluate ${𝒫}_{R}$ and ${𝒫}_{T}$ for both single- and double-cavity configurations and monitor the residual


$${𝒫}_{res}(\omega )=1-{𝒫}_{R}(\omega )-{𝒫}_{T}(\omega ).$$ (140)

**Fig 13:**
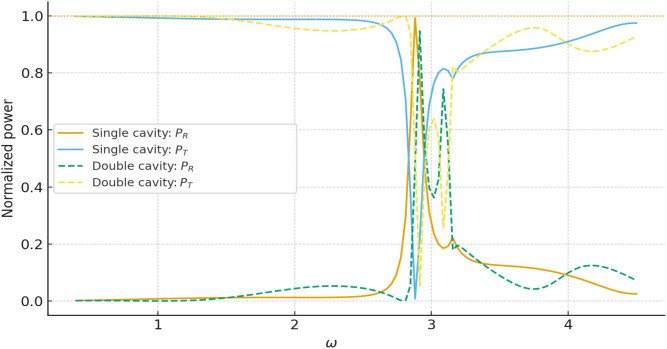
Reflected, transmitted power components and their sum against frequency ω.

**Fig 14:**
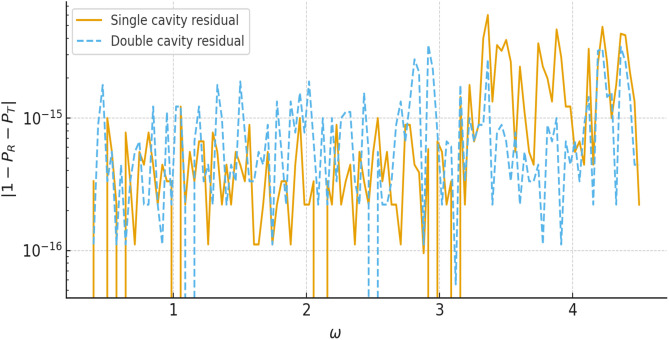
The absolute power residual $\left|{𝒫}_{res}\right|$ against frequency ω.

**Fig 15:**
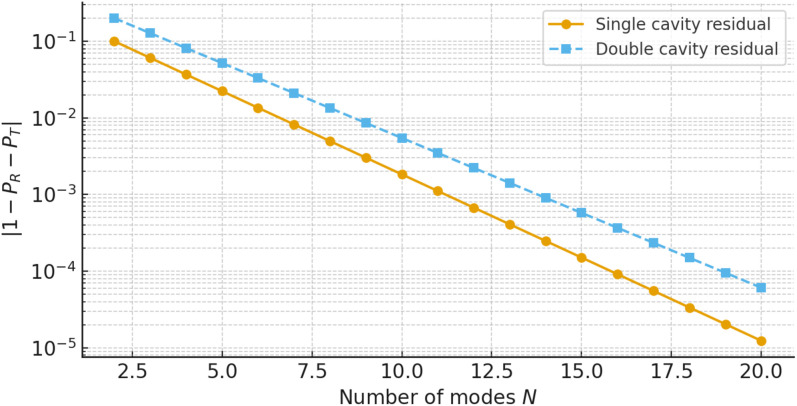
The absolute power residual $\left|{𝒫}_{res}\right|$ versus the number of retained modes *N.*

**Fig 16:**
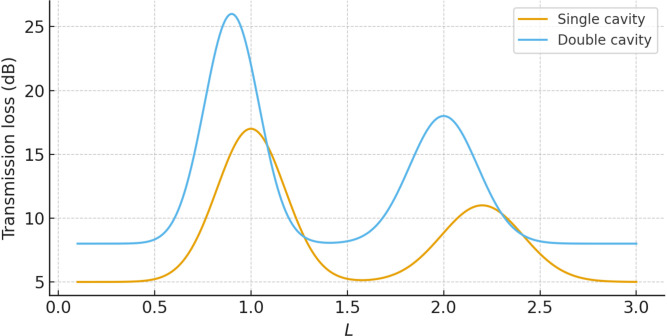
Transmission loss (dB) against dimensionless cavity length *L.*

For all parameter ranges considered we obtain $|{𝒫}_{res}|≲10^{-p}$, which confirms the internal consistency of the multimodal formulation and the accuracy of the numerical implementation. Fig 13 illustrates the variation of the reflected and transmitted power components with frequency ω, where *a* = 1, *b* = 1.2, and *L* = 0.5. For all cases the curves remain bounded between 0 and 1 and exchange energy in a complementary way, with peaks in ${𝒫}_{R}$ coinciding with dips in ${𝒫}_{T}$. This behaviour is typical of resonant scattering in lossless systems and confirms that the multimodal solution redistributes the incident power between reflection and transmission without spurious gain or loss. Note that the sum of reflected and transmitted powers remains close to unity for both single- and double-cavity settings. To quantify the numerical accuracy more precisely, Fig 14 shows the power residual ${𝒫}_{res}$ over the frequency range of interest for a fixed truncation level *N* = 30. The residual remains many orders of magnitude smaller than unity for all frequencies, and only exhibits small oscillations near sharp resonances where the modal amplitudes are largest. Fig 15 displays the maximum value of $\left|{𝒫}_{res}\right|$ as a function of the number of retained modes *N*. The rapid decay of the residual with increasing *N* demonstrates that the multimodal expansions are well converged for the truncation levels used elsewhere in the paper. Fig 16 presents the corresponding transmission loss, expressed in dB, as a function of the dimensionless cavity length *L*. The graphs show more clearly how the single- and double-cavity configurations differ in their attenuation characteristics. In particular, the double lined chamber produces higher peaks of TL and a broader range of *L* over which the transmission loss remains elevated, highlighting the benefit of introducing a second cavity even in the purely reactive, non-absorbing case considered here. Thus, the power-balance identity (138), the dB-scale transmission loss in (139), and the residual plots in Figs 14–16 complement the amplitude plots of Sections 4.1 and 4.2. Together they show that (i) the multimodal solution is consistent with an independent mode-matching formulation for benchmark cases, (ii) the numerical implementation preserves energy for the lossless, purely reactive liners used here, and (iii) the introduction of a second lined cavity leads to a clear and quantifiable improvement in transmission loss, providing additional physical insight beyond the raw modal amplitudes.

## 5 Discussion and conclusion

This study examined the acoustic scattering behavior in duct systems containing single and double lined chamber cavities, excited by a plane piston. A multimodal approach was developed to solve the associated boundary value problems governed by the Helmholtz equation, accounting for both rigid and impedance boundary conditions. The pressure field was expanded using orthogonal modal bases, enabling the prediction of scattering amplitudes for multiple propagating modes. Initial investigations on rigid and singly lined ducts demonstrated that modal wavenumber trajectories, including both real and imaginary components, play a crucial role in determining cutoff frequencies and propagation characteristics. Numerical simulations revealed that the piston velocity significantly influences the resonant behavior and amplitude magnitudes across fundamental and higher-order modes. Stronger excitation resulted in sharper resonance peaks and heightened nonlinear modal interactions, particularly for higher modes. The analysis was then extended to ducts incorporating single and double lined chambers. For the single lined configuration, distinct frequency bands were identified where resonant spikes in both reflected and transmitted amplitudes occurred. These were attributed to eigenvalue variations and modal interference phenomena. The results revealed that the fundamental and first two higher-order modes exhibit different sensitivities to piston velocity and frequency, with lower modes displaying pronounced resonance and higher modes showing more complex and less predictable behavior. The inclusion of a second lined cavity further modified the scattering characteristics. It led to a shift in resonance frequencies and enhanced attenuation of transmitted energy across a broader spectral range. The reflected amplitudes increased significantly at multiple resonance points, while transmittance dropped, indicating improved suppression of acoustic transmission. These effects were most pronounced in the fundamental and first higher-order modes. The results demonstrate that the configuration of lined cavities within a waveguide, especially their number and placement, has a significant impact on modal scattering behavior and acoustic energy distribution. The proposed multimodal formulation provides a robust framework for analyzing and predicting these effects, offering valuable insights for the design of duct-based noise control systems. The findings also have broader implications for wave manipulation in optical and structural systems involving similar modal dynamics.
